# Supplementary material for: Taste sensing and sugar detection mechanisms in Drosophila larval primary taste center
Source: eLife. 2021 Dec 3;10:e67844. doi: 10.7554/eLife.67844 (PMC8709573; doi:10.7554/eLife.67844)
Supplement: Figure 1—figure supplement 1—source data 1. — CaImg_analysis_pipeline contains ImageJ scripts for macro/plugin and instructions. [file elife-67844-fig1-figsupp1-data1.zip › CaImg_analysis_pipeline/plugin/stack-tool_duplication_plugin_readme.rtf]

ImageJ plugin for single time-point signal duplication onto time-series stack.
Also see: https://imagej.net/Plugins

Workflow:
- single time-point stack in ImageJ - to be duplicated as the first channel of the resulting image for all time-points
- time-series stack in ImageJ - will represent the second channel of the resulting image, unchanged on time axis

	1.	open both the reference stack and the time series recording in Fiji-ImageJ 
	2.	select Time-Series plugin Merge1+T
	3.	in the dropdown menu choose the corresponding file names for the reference stack and the time series
	4.	click ok
