# Supplementary material for: Taste sensing and sugar detection mechanisms in Drosophila larval primary taste center
Source: eLife. 2021 Dec 3;10:e67844. doi: 10.7554/eLife.67844 (PMC8709573; doi:10.7554/eLife.67844)
Supplement: Figure 1—figure supplement 1—source data 1. — CaImg_analysis_pipeline contains ImageJ scripts for macro/plugin and instructions. [file elife-67844-fig1-figsupp1-data1.zip › CaImg_analysis_pipeline/README_live_imaging_data_processing_pipeline.rtf]

Data processing pipeline designed for live imaging with input and desired output result:OUTPUT: stable hyperstack of 2-channel image over time. INPUT: 2 different signals acquired as separate recordings: 	1. single stack serving as reference signal (e.g. fluorescence signal from cell nuclei for efficient segmentation) - in this study: nuclear RFP in sensory neurons	2. time-series recording cell data over time on the same sample; could contain movement associated with live imaging on tissue/living animal (e.g. cytoplasmic fluorescence over time for the same cells as recorded in the reference stack)- in this study: cytoplasmic GCaMP fluorophore in sensory neuronsPipeline steps:Step 1. 3D drift correction for the time-series recording using ImageJ plugin (https://imagej.net/Correct_3D_Drift). If stable recording, this step can be skipped and stabilisation can be performed directly on the final two-channel result.Step 2. Reference stack duplication over all time points of the time series using the stack-tools-1.1.1-SNAPSHOT.jar found in plugin folder enclosed here. Read README instruction file at the same location for details. Step 3. Correction of misalignment between the two merged signals of a live tissue recording using Time_Series-Alignment.py found in the script_macro folder enclosed here. Read README instruction file at the same location for details. Step 4. On the resulting 2-channel recording with aligned signal a final 3D drift correction can be applied to obtain a steady time-series.
