# Supplementary material for: Taste sensing and sugar detection mechanisms in Drosophila larval primary taste center
Source: eLife. 2021 Dec 3;10:e67844. doi: 10.7554/eLife.67844 (PMC8709573; doi:10.7554/eLife.67844)
Supplement: Figure 1—figure supplement 1—source data 1. — CaImg_analysis_pipeline contains ImageJ scripts for macro/plugin and instructions. [file elife-67844-fig1-figsupp1-data1.zip › CaImg_analysis_pipeline/script_macro/time-series_alignment_readme.rtf]

ImageJ script for assisted manual alignment correction between two channels.
Also see: https://imagej.net/Scripting

Workflow:
- select a reference point on a frame with misaligned channels, then select a second point to mark the displaced center of the object. The translation vector between the 2 points will be calculated and applied.
- selection points have to be in the same or in adjacent time-frames and they have to be set in pairs.

1.	open the 2-channel recording in Fiji/ImageJ
2.	open Time_Series-Alignment.py in Fiji/ImageJ
3.	click run	
4.	select Propagate transform if the correction should apply for all the frames 	following the selected frame
5.	click ok
6.	add first selection point on one region in the channel to be corrected
7.	either in the same or in the following time-frame,  add second selection point on 	the reference channel corresponding to the same region as in step 6
8.	click ok
9.	the correction is applied for one frame or for all following time-points if the 	Propagate transform option was active
10.	repeat the process for as many time points as needed
