# Supplementary material for: Taste sensing and sugar detection mechanisms in Drosophila larval primary taste center
Source: eLife. 2021 Dec 3;10:e67844. doi: 10.7554/eLife.67844 (PMC8709573; doi:10.7554/eLife.67844)
Supplement: Transparent reporting form [file elife-67844-transrepform1.docx]

***eLife’s* transparent reporting form**

We encourage authors to provide detailed information *within their submission* to facilitate the interpretation and replication of experiments. Authors can upload supporting documentation to indicate the use of appropriate reporting guidelines for health-related research (see [EQUATOR Network](http://www.equator-network.org/%20)), life science research (see the [BioSharing Information Resource](https://biosharing.org/)), or the [ARRIVE guidelines](http://www.plosbiology.org/article/info:doi/10.1371/journal.pbio.1000412) for reporting work involving animal research. Where applicable, authors should refer to any relevant reporting standards documents in this form.

If you have any questions, please consult our Journal Policies and/or contact us: [editorial@elifesciences.org](mailto:editorial@elifesciences.org).

**Sample-size estimation**

- You should state whether an appropriate sample size was computed when the study was being designed
- You should state the statistical method of sample size computation and any required assumptions
- If no explicit power analysis was used, you should describe how you decided what sample (replicate) size (number) to use

Please outline where this information can be found within the submission (e.g., sections or figure legends), or explain why this information doesn’t apply to your submission:

Calcium imaging recordings: sample size stated in legend of Figure 2 and Figure2-SourceData1 and 2, Results section “**Sucrose elicits neuronal responses in the TOG”** and Methods section Calcium Imaging.

Behavior tests: sample size is mentioned in legend of Figure 4, Figure4 – Figure Supplement 1 and the respective Source Data files and in Methods section on larval behavior.

**Replicates**

- You should report how often each experiment was performed
- You should include a definition of biological versus technical replication
- The data obtained should be provided and sufficient information should be provided to indicate the number of independent biological and/or technical replicates
- If you encountered any outliers, you should describe how these were handled
- Criteria for exclusion/inclusion of data should be clearly stated
- High-throughput sequence data should be uploaded before submission, with a private link for reviewers provided (these are available from both GEO and ArrayExpress)

Please outline where this information can be found within the submission (e.g., sections or figure legends), or explain why this information doesn’t apply to your submission:

We considered biological replicates as measurements or recordings on distinct biological preparations/animals in consecutive runs during the same experimental conditions.

To account for technical variability in measurement tools, protocol or substance solutions we repeated experiments by reproducing test conditions at different times for technical replicates.

All data in this study consists of both biological and technical replicates according to this definition.

**Statistical reporting**

- Statistical analysis methods should be described and justified
- Raw data should be presented in figures whenever informative to do so (typically when N per group is less than 10)
- For each experiment, you should identify the statistical tests used, exact values of N, definitions of center, methods of multiple test correction, and dispersion and precision measures (e.g., mean, median, SD, SEM, confidence intervals; and, for the major substantive results, a measure of effect size (e.g., Pearson's r, Cohen's d)
- Report exact p-values wherever possible alongside the summary statistics and 95% confidence intervals. These should be reported for all key questions and not only when the p-value is less than 0.05.

Please outline where this information can be found within the submission (e.g., sections or figure legends), or explain why this information doesn’t apply to your submission:

Statistical analysis was used for behaviour tests as indicated in legend of Figure 4 and the respective source data.

(For large datasets, or papers with a very large number of statistical tests, you may upload a single table file with tests, Ns, etc., with reference to sections in the manuscript.)

**Group allocation**

- Indicate how samples were allocated into experimental groups (in the case of clinical studies, please specify allocation to treatment method); if randomization was used, please also state if restricted randomization was applied
- Indicate if masking was used during group allocation, data collection and/or data analysis

Please outline where this information can be found within the submission (e.g., sections or figure legends), or explain why this information doesn’t apply to your submission:

For calcium imaging recordings order of substances for stimulation was randomized.

**Additional data files (“source data”)**

- We encourage you to upload relevant additional data files, such as numerical data that are represented as a graph in a figure, or as a summary table
- Where provided, these should be in the most useful format, and they can be uploaded as “Source data” files linked to a main figure or table
- Include model definition files including the full list of parameters used
- Include code used for data analysis (e.g., R, MatLab)
- Avoid stating that data files are “available upon request”

Please indicate the figures or tables for which source data files have been provided:

***Figure 1 – Figure Supplement 1***: ImageJ plugin and macro for calcium imaging data processing with instructions

***Figure 2 – Source data 1*** for graphs B & C and ***Figure 2 – Source data 2*** for panel D/D’ as excel sheets comprising neuronal calcium imaging data and lists of neuronal responses

***Figure 3 – Source data 1*** for bar plots in Fig.3C as excel file, comprising responses associated with mapped cells

***Figure 4 – Source data 1*** for Fig.4A, B, C as excel file containing larval preference index used for the figure generation

***Figure 4 – Figure Supplement 1 – Source data 1***: for Fig.4-Fig.Suppl1A and B containing larval preference index referred to in this figure
